# Supplementary material for: m6a methylation orchestrates IMP1 regulation of microtubules during human neuronal differentiation
Source: Nat Commun. 2024 Jun 6;15:4819. doi: 10.1038/s41467-024-49139-7 (PMC11156911; doi:10.1038/s41467-024-49139-7)
Supplement: Supplementary file 1 — Supplementary Information [file 41467_2024_49139_MOESM1_ESM.pdf]

Supplemental material to the manuscript:

m6a methylation orchestrates IMP1 regulation of microtubules during human neuronal differentiation

Pierre Klein[1,2], Marija Petrić Howe[2,3], Jasmine Harley[2,3], Harry Crook[2], Sofia Esteban Serna[1], Theodoros I. Roumeliotis[4], Jyoti S. Choudhary[4], Anob M Chakrabarti[5], Raphaëlle Luisier[6,7], Rickie Patani[2,3]*, Andres Ramos[1]*

[1]Division of Biosciences, Institute of Structural and Molecular Biology University College London, Darwin Building, Gower Street, London, WC1E 6XA, UK
[2]Human Stem Cells and Neurodegeneration Laboratory, The Francis Crick Institute, 1 Midland Road, London NW1 1AT, UK
[3]Department of Neuromuscular Diseases, Queen Square Institute of Neurology, University College London, London WC1N 3BG, UK
[4]Functional Proteomics team, The Institute of Cancer Research, 237 Fulham Road, London SW3 6JB, UK
[5]RNA Networks Laboratory, The Francis Crick Institute, 1 Midland Road, London NW1 1AT, UK
[6]Idiap Research Institute, Martigny 1920, Switzerland
[7]SIB Swiss Institute of Bioinformatics, Lausanne 1015, Switzerland
Correspondence to a.ramos@ucl.ac.uk and rickie.patani@ucl.ac.uk

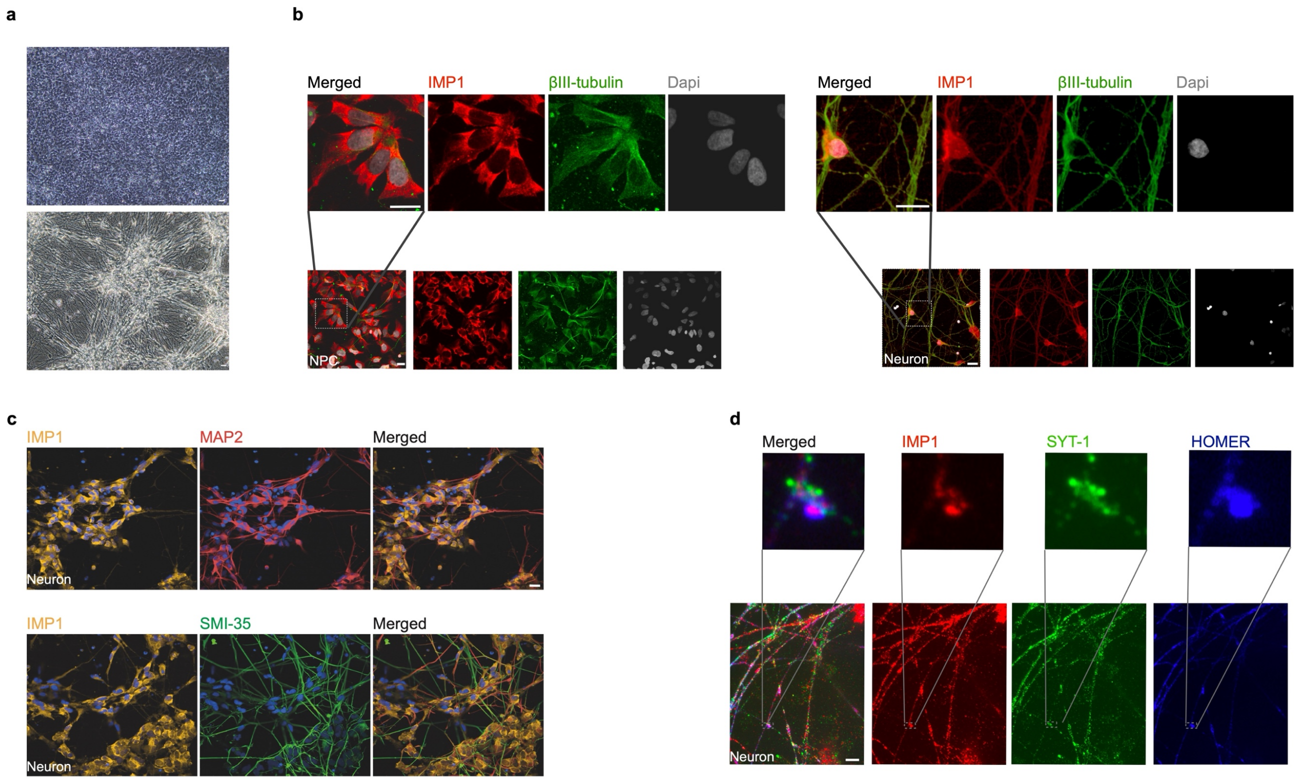

Supplementary Fig.1 - IMP1 expression in neurites and synapses of differentiating neurons (related to Figure 1)

a, Representative light microscopy images of neural precursor cells - NPCs (top) and neurons (bottom) used in this study. Scale bar, 20 µm. Three independent iPSC lines were used for both NPCs and neurons (biological replicates).

b, Representative confocal images of NPCs (left) and neurons (right) immunolabeled for IMP1 (red) or βIII-tubulin (green); nuclei counterstained with DAPI (grey). Scale bar, 20 µm.

c, Representative confocal images of neurons immunolabeled for IMP1 (yellow) and MAP2 (red); nuclei counterstained with DAPI (blue) (top row); IMP1 (yellow), SMI-35 (green) and nuclei counterstained with DAPI (blue) (bottom row). Scale bar, 20 µm.

d, Representative instantaneous super-resolution (VT-iSIM) image of neurons immunolabeled for IMP1 (red), presynaptic marker SYT-1 (green) and postsynaptic marker Homer-1 (HOMER) (blue). Scale bar, 10 µm.

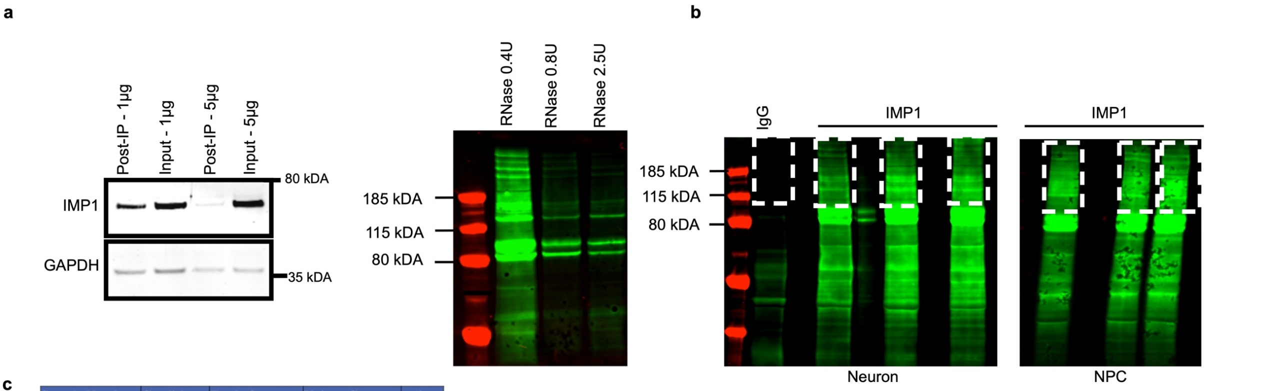

c

Sample Number	Condition	Total of counts	Unique Counts	ratio
	IgG	1.40E+05	7.71E+04	1.82
1	Neuron	6.28E+06	3.48E+06	1.81
2	Neuron	2.87E+07	6.95E+06	4.13
3	Neuron	1.41E+07	3.54E+06	3.97
4	Neuron	9.07E+06	5.03E+06	1.81
5	Neuron	6.50E+06	4.22E+06	1.54
6	Neuron	8.48E+06	2.74E+06	3.09
7	Neuron	2.59E+07	4.95E+06	5.22
8	Neuron	8.46E+06	4.99E+06	1.70
9	Neuron	9.38E+06	4.67E+06	2.01
**Sample Number**	**Condition**	**Total of counts**	**Unique Counts**	**ratio**
1	NPC	2.36E+07	6.56E+06	3.59
2	NPC	6.19E+06	2.47E+06	2.51
3	NPC	8.22E+06	4.34E+06	1.90
4	NPC	5.94E+06	2.42E+06	2.45
5 *	NPC	1.86E+06	3.27E+05	5.67
6	NPC	7.03E+06	2.35E+06	2.99
7	NPC	5.14E+06	1.74E+06	2.95
8	NPC	7.85E+06	1.28E+06	6.13
9 *	NPC	6.28E+03	2.47E+03	2.54

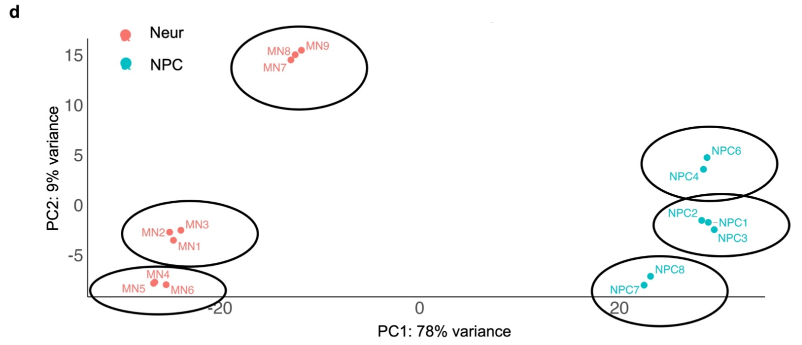

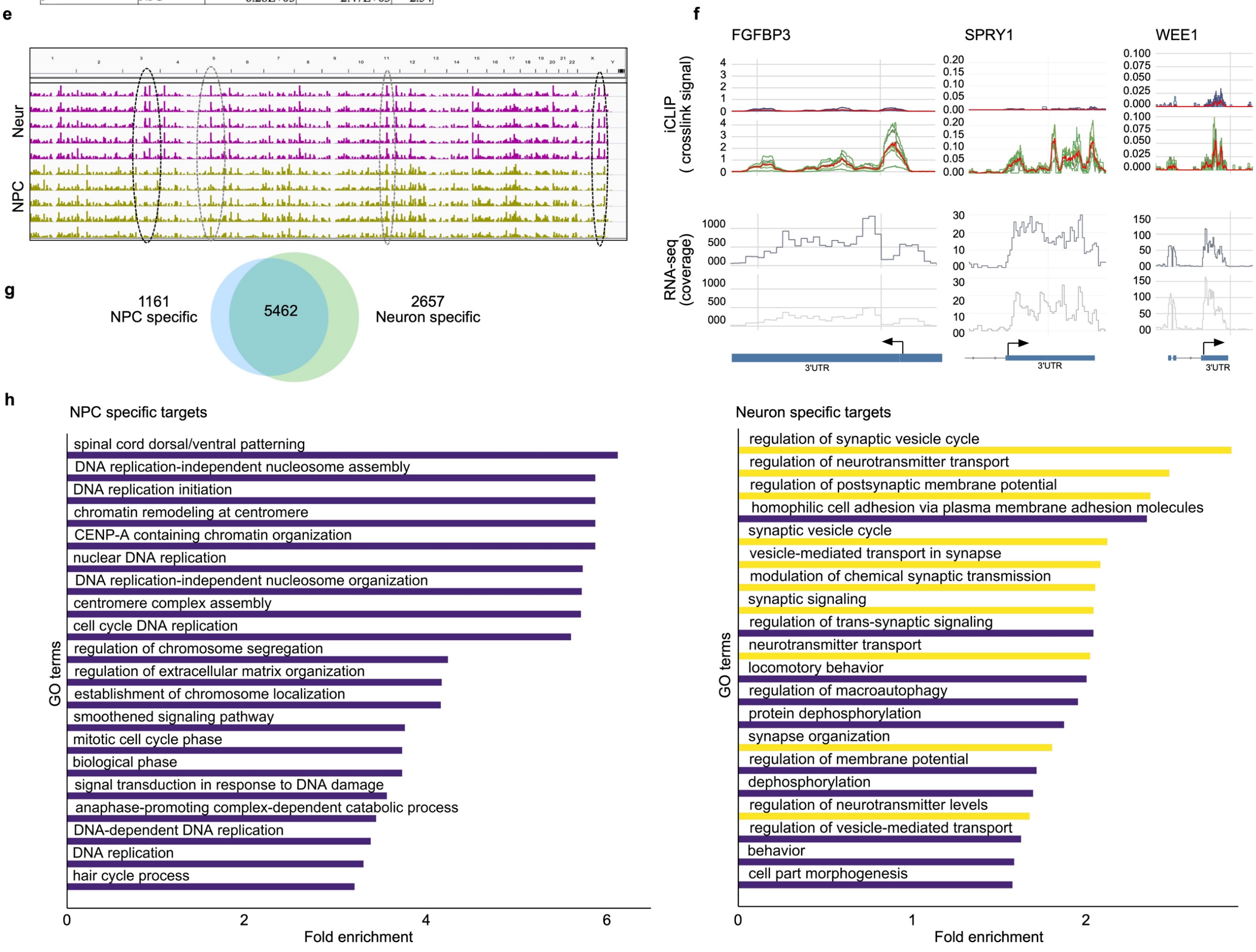

Supplementary Fig.2 - iCLIP captures IMP1-RNA binding in NPCs and neurons (related to Figures 1 and 2)

a, Left - Western blot validation of IMP1 immunoprecipitation antibody concentration optimization for the iCLIP experiment. A concentration of 1µg and 5µg of antibody per 1 mg of

protein lysate was tested. GAPDH immunostaining was used as a loading control. Right - representative LI-COR scanning visualisation of RNA/IMP1 complexes on a nitrocellulose membrane in neurons treated with a gradient of RNAse concentrations - 0.4, 0.8 and 2.5U per 1 mL of lysate at a protein concentration of 1 mg/ml. Visualisation is based on the infrared adaptor ligated to RNA within protein-RNA complexes.

b, Representative LI-COR scanning visualisation of RNA/IMP1 or RNA/IgG complexes in neurons and NPCs on a nitrocellulose membrane. Visualisation is based on the infrared adaptor ligated to RNA within protein-RNA complexes. The portion of the membrane excised and used to generate iCLIP libraries is shown in dashed white lines.

c, Table indicating the number of total counts (reads), unique counts and ratio of both for each iCLIP sample. IgG condition was used as a negative technical control. Red asterisks show samples excluded from further analysis because of a significantly lower number of unique counts compared to other samples.

d, Principal component analysis (PCA) of cDNA counts from each neuron and NPC sample iCLIP experiment. Technical replicates for each clone are circled.

e, Mapping tracks of iCLIP crosslink signals on the whole transcriptome, visualised using Integrative Genomics Viewer software for five neuronal and five NPC samples, in pink and yellow respectively. Dotted ovals highlight similar (light grey) and dissimilar (dark grey) genomic crosslink regions between NPCs and neurons.

f, IMP1 iCLIP crosslinks (counts per millions) and RNAseq data (reads coverage) are mapped onto genomic coordinates in NPCs and neurons for 3 exemplar target mRNAs. iCLIP replicates are shown in blue for neurons and in green for NPCs, while the merged signal is in red. 3' untranslated region (3'UTR) and last exon are marked by a blue box and separated by an arrow, while line arrows indicate other intronic boundaries. FGFBP3 serves as an illustrative example of a gene displaying decreased IMP1 binding in neurons, coupled with increased expression when compared to NPCs. In contrast, SPRY1 and WEE1 represent instances of decreased IMP1 binding, yet their expression levels remain unchanged in neurons compared to NPCs.

g, Venn diagram representing the overlap between transcripts bound by IMP1 in neurons and NPCs. When considering the expression of the 2657 neuron-specific genes, 1218 are up-regulated and 722 are down-regulated.

h, Top 20 significantly enriched GO terms of genes that IMP1 uniquely binds in neurons or NPCs, as defined using PANTHER. Terms are ranked based on fold enrichment. All terms presented have a false discovery rate (FDR) <0.05 and p-value <0.01. P-value was calculated using Fisher's Exact test. Yellow bars highlight terms related to neurogenesis pathways. Source data are provided as a Source Data file

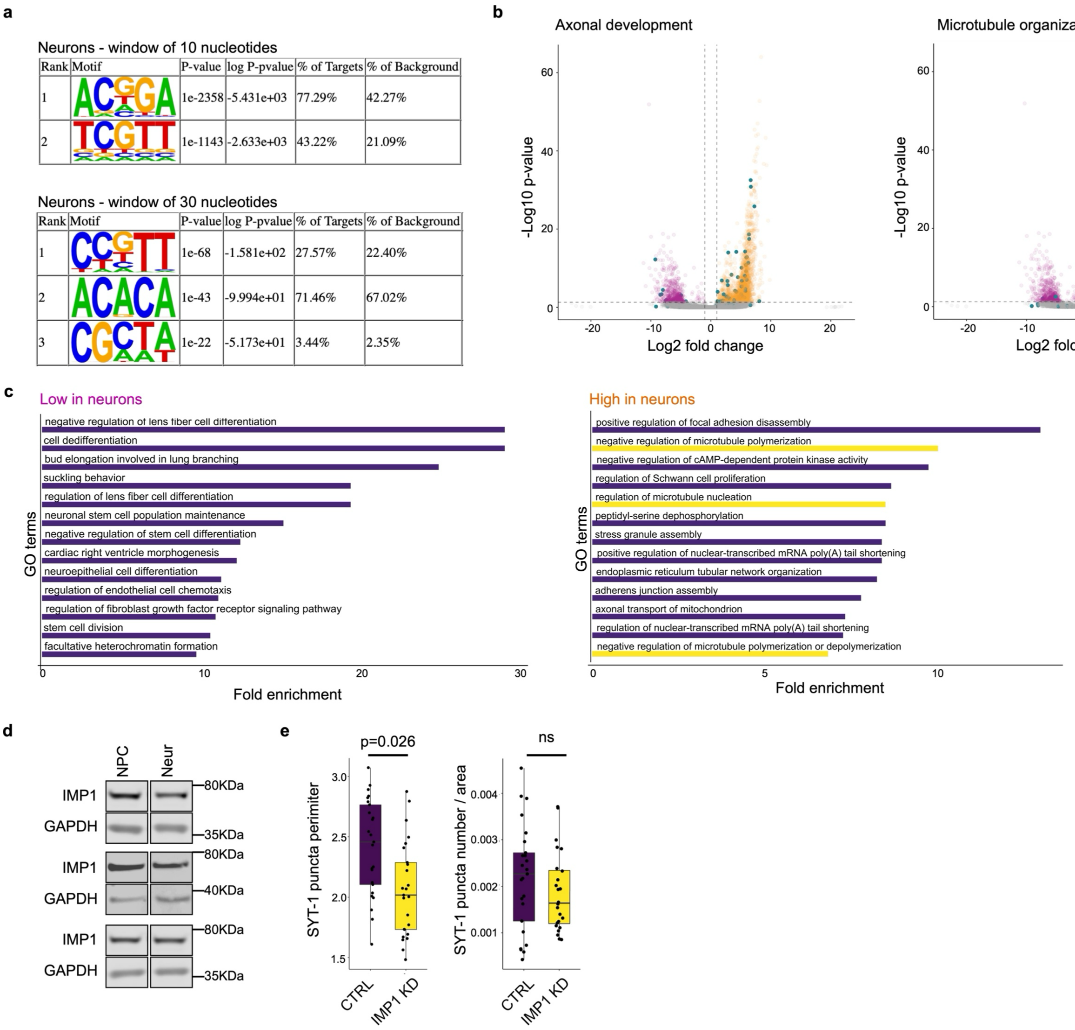

Supplementary Fig.3 - IMP1 reconfigures its RNA targetome to neuronal mRNAs and regulates synaptic puncta during development (related to Figure 2)

a, Top most significantly enriched de novo sequence motifs within IMP1 binding sites in its neuronal 3'UTR targets determined by HOMER. Motif analysis was performed for a window of 10 or 30 nucleotides around IMP1 binding sites. The p-values of the motifs, % of targets and % of background are shown. P-value was calculated using a binomial distribution test.

b, Volcano plot of IMP1 bound individual peaks normalized by gene expression changes in NPC vs neurons, related to Fig. 2b. Peaks with significantly higher signal in NPCs vs neurons, and vice versa are shown respectively in pink and orange. P-values were calculated using a likelihood-ratio test (LRT). A threshold of 1 < log2 FC < -1 and adjusted p-value < 0.05 was used to determine significance. The vertical dashed lines mark log2 FCs < - 1 or >1, while the horizontal dashed line marks an adjusted p-value < 0.05. Blue dots highlight peaks belonging to microtubule organization (GOs: regulation of cytoskeleton organization, microtubule-based process) or axonal development (GOs: neuron projection development, modulation of synaptic transmission, synaptic vesicle cycle) genes. The ensemble of high-in-neuron (orange) peaks is 3.3 times larger than that in high-in-NPC (pink) peaks. When we consider only axonal development terms the ratio between high-in-neuron and high-in-NPC is 4.5, while when microtubule organization terms are considered, the ratio is instead of 8.1. Please note the position of individual dots (including blue dots) is often overlapped.

c, Top GO terms by enrichment of IMP1 bound genes normalized by gene expression changes in NPCs vs neurons - related to Figure 2b. On the left are displayed terms comprised by genes that exhibit low IMP1 binding, on the right terms comprised by genes highly bound by IMP1. Terms are ranked based on fold enrichment. All terms presented have a false

discovery rate (FDR) <0.05. P-value was calculated using Fisher's Exact test. Yellow bars highlight terms related to microtubule regulation.
d, Representative Western blot images showing expression of IMP1 and GAPDH as loading control in NPCs and neurons, used for quantification in Figure 2c.
e, Left - quantification of SYT-1 puncta perimeter (pixels) in neurons treated with IMP1 siRNA (IMP1) vs non-targeting control (CTRL). Data presented as boxplots where the center line is the median, limits are the interquartile range and whiskers correspond to 1.5 times the interquartile range. Outliers are not displayed for clarity. Data points represent different fields of view, n=2 independent iPSC lines (biological replicates) in 2 independent experiments. P- value was calculated using an unpaired two-sided t-test and reported on the plot. Right - quantification of the number of SYT-1 puncta per area of βIII-tubulin positive neurites treated with non-targeting control siRNA (CTRL) or siRNA targeting IMP1 (IMP1). Data presented as boxplots where the center line is the median, limits are the interquartile range and whiskers correspond to 1.5 times the interquartile range. Outliers are not displayed for clarity. Data points represent different fields of view, n=2 independent iPSC lines (biological replicates) in 2 independent experiments. P-value calculated using two-sided Mann–Whitney U test indicates the difference is not significant (annotated as ‘ns’).
Source data are provided as a Source Data file.

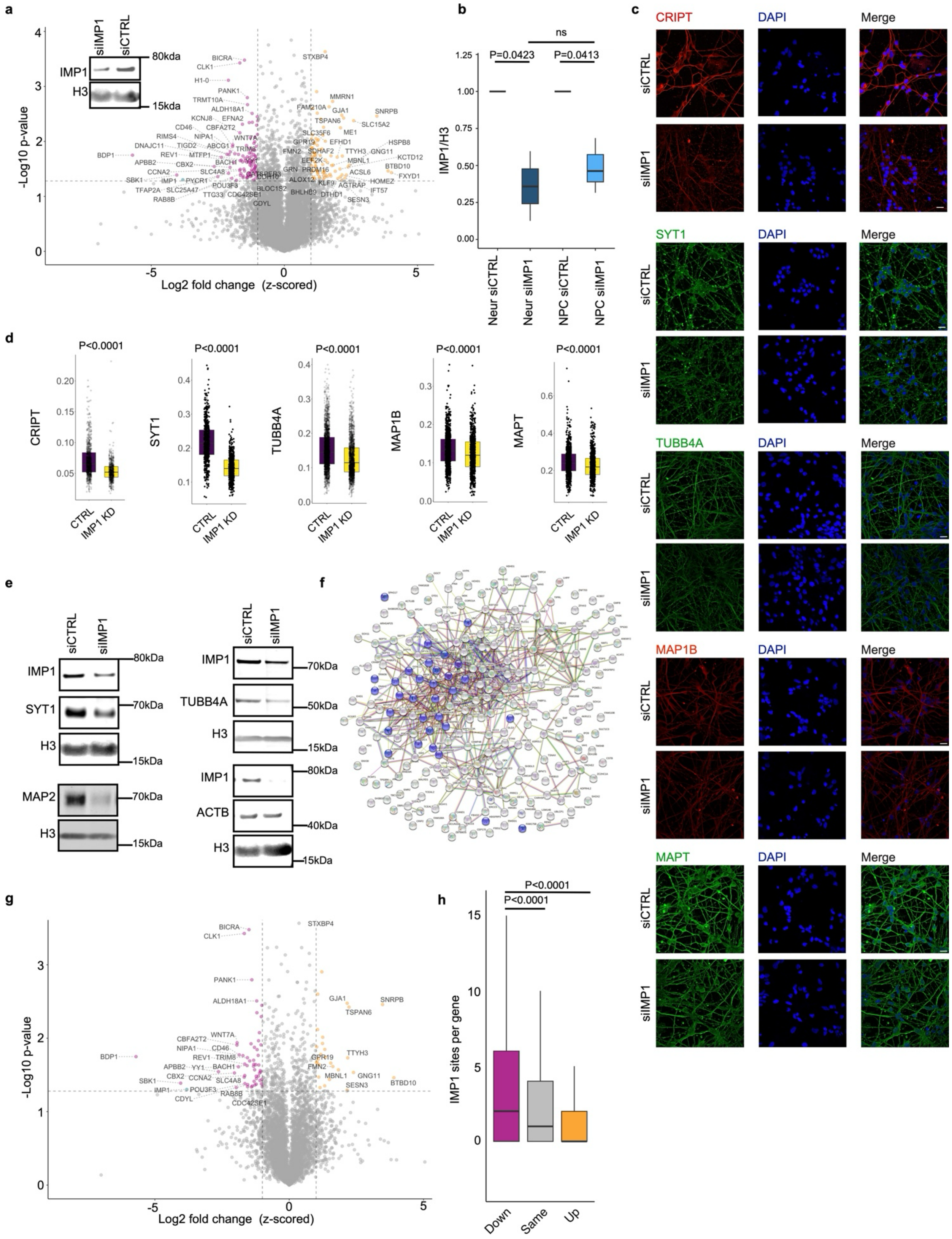

Supplementary Fig.4 - IMP1 regulates protein abundance of directly bound mRNA targets in NPCs and neurons (related to Figures 3 and 4)

a, Volcano plot of proteins differentially expressed in IMP1 siRNA (siIMP1) vs non-targeting control (siCTRL) NPCs. Vertical dashed lines indicate log2 FC significance thresholds of 1 and -1, z-scored, while the horizontal dashed line denotes a p-value significance threshold of 0.05. The proteins significantly up- or down-regulated are highlighted in orange and pink respectively. A two-sided one-sample Student's t-test was used on n = 2 independent iPSC lines for each condition (biological replicates). The inset depicts a representative Western blot demonstrating

IMP1 protein silencing, with H3 as loading control, n=3 independent iPSC lines (biological replicates).

b, Relative expression of IMP1 over H3 measured by Western blotting for IMP1 and control siRNA treated NPC and neuron samples used in the mass spectrometry analysis. Data presented as boxplots where the center line is the median, limits are the interquartile range and whiskers correspond to 1.5 times the interquartile range. Outliers are not displayed for clarity. n=3 and 2 independent iPSC lines (biological replicates) for neurons and NPCs respectively. P-values were calculated using a two-sided Mann–Whitney U test and displayed on the plot. ns indicates that the difference is not significant. IMP1 siRNA treated samples are normalized on relative expression of corresponding clones treated with siRNA control.

c, Representative confocal images of neurons treated with siRNA control or siRNA IMP1 and immunolabeled for CRIPT, SYT1, TUBB4A, MAP1B or MAPT and counterstained with DAPI. Scale bar, 20 µm.

d, Quantification of CRIPT, SYT1, TUBB4A, MAP1B, MAPT protein expression from immunostaining of neurons treated with non-targeting control siRNA (CTRL) or IMP1 siRNA (IMP1 KD). Each point represents an individual cell cytoplasmic intensity. Data presented as boxplots where the center line is the median, limits are the interquartile range and whiskers correspond to 1.5 times the interquartile range. Outliers are not displayed for clarity. n=3 independent iPSC lines (biological replicates) in 3 independent experiments. P-values were calculated using two-sided Mann–Whitney U test, all ranging at <0.0001.

e, Representative Western blot images showing expression of IMP1, SYT1, MAP2, TUBB4A, ACTB, and H3 as loading control in neurons treated with non-targeting control siRNA (siCTRL) or siRNA targeting IMP1 (siIMP1).

f, STRING analysis of the protein–protein interaction network of proteins downregulated in IMP1 siRNA treated neurons (log2 FC < -1, P < 0.05). Proteins that belong to the STRING GO term “microtubule-based process” are highlighted in blue. Network protein-protein interaction (PPI) enrichment p-value < 1.0e-16.

g, Volcano plot of differentially expressed proteins in NPCs treated with IMP1 siRNA compared to non-targeting control (detected by MS) for IMP1 bound transcripts only (detected by iCLIP). Downregulated, unchanged and upregulated proteins are shown in pink, grey and orange respectively. For downregulated proteins selection was based on log2 FC < -1, p-value < 0.05, two-sided one-sample Student’s t-test, for upregulated proteins selection was based on log2 FC > 1, p-value < 0.05, two-sided one-sample Student’s t-test, for transcripts containing at least one IMP1 binding site. For MS, n=2 independent iPSC lines for each condition (biological replicates); for iCLIP, n=2 technical replicates from 2 independent iPSC lines (biological replicates) + 3 technical replicates from 1 independent iPSC line.

h, Number of IMP1 peaks per gene detected by iCLIP for proteins downregulated, unchanged or upregulated in response to IMP1 knockdown, log2 FC < -1, -1 > log2 FC < 1, log2 FC > 1 respectively (see Fig. 3a). Data are presented as boxplots where the center line is the median, limits are the interquartile range and whiskers correspond to 1.5 times the interquartile range. Outliers are not displayed for clarity. For MS, n=3 independent iPSC lines for each condition (biological replicates); for iCLIP, n=3 technical replicates from 3 independent iPSC lines (biological replicates). P-values were calculated using Kruskal–Wallis with Dunn’s multiple comparisons test, all ranging at <0.0001.

Source data are provided as a Source Data file.

a

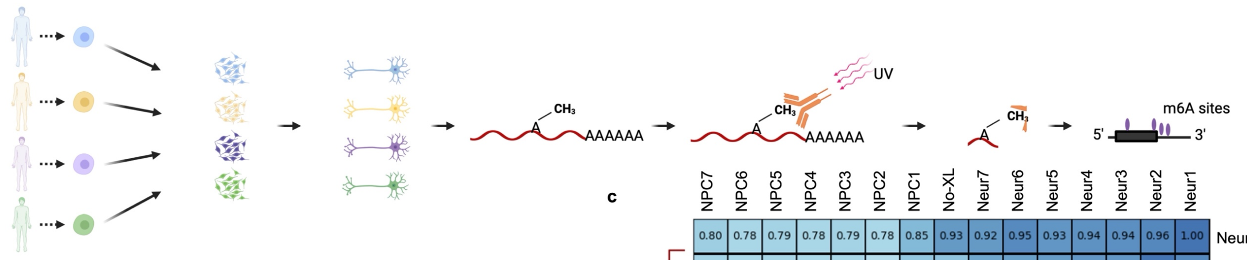

b

Sample Number	Condition	Total of counts	Unique Counts	ratio
	no_XL	5.05E+06	2.51E+06	2.01
1	Neuron	2.21E+06	3.52E+05	6.29
2	Neuron	5.50E+06	2.40E+06	2.29
3	Neuron	1.01E+07	4.82E+06	2.11
4 *	Neuron	3.00E+06	2.58E+05	11.61
5	Neuron	7.82E+06	2.70E+06	2.89
6	Neuron	2.50E+06	1.31E+06	1.90
7	Neuron	2.62E+07	1.05E+07	2.49
8	Neuron	2.84E+07	1.15E+07	2.48
**Sample Number**	**Condition**	**Total of counts**	**Unique Counts**	**ratio**
1 *	NPC	1.63E+05	1.60E+04	10.17
2	NPC	4.00E+06	2.58E+05	15.48
3	NPC	2.02E+06	8.90E+05	2.27
4	NPC	1.95E+06	4.99E+05	3.90
5	NPC	2.90E+06	1.21E+06	2.40
6	NPC	2.24E+06	1.06E+06	2.12
7	NPC	4.63E+06	1.53E+06	3.03
8	NPC	7.31E+06	2.35E+06	3.12

c

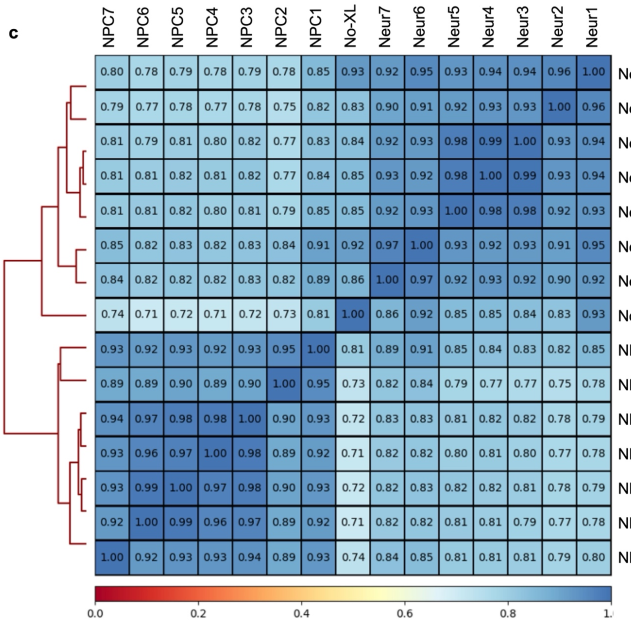

d

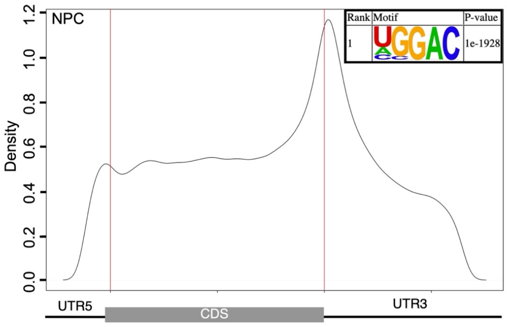

e

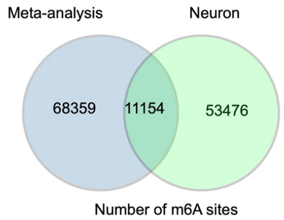

f

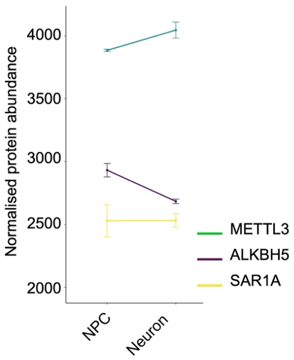

g

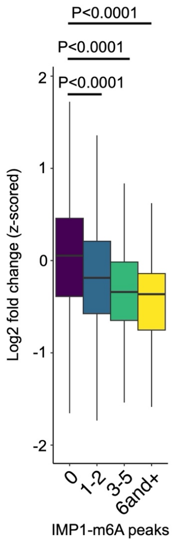

h

Category	Transcript	Peak
IMP1 bound m6A targets/IMP1 targets	14.3%	4.7%
IMP1 bound m6A targets regulated at protein level /IMP1 targets regulated at protein level	31.7%	7.4%

Supplementary Fig.5 - m6A methylation and related IMP1 binding increase during neuronal development (related to Figure 5)

a, Schematic showing miCLIP strategy in NPCs and neurons. Briefly, poly(A)+ RNA was extracted, and UV-C crosslinked to an m6A antibody (m6A ab). RNA was then fragmented by

RNase I, m6A ab-mRNA complexes were immunoprecipitated (IP) and RNA was linked to an infrared dye-labeled oligo. Complexes were run on an SDS-PAGE and transferred to a nitrocellulose membrane. RNA was visualised using the IR dye and the region of interest was released from the membrane by proteinase K digestion. RNA was reverse transcribed, circularised, and PCR amplified. Two technical replicates from four independent iPSC lines were used for NPCs and neurons (biological replicates). Panel created with BioRender.com released under a Creative Commons Attribution-NonCommercial-NoDerivs 4.0 International license.

b, Table indicating the number of total counts (or reads), unique counts and ratio of both for each miCLIP sample. Non crosslinked and IgG conditions were used as negative technical controls (only the non-crosslinked condition was sequenced due to the absence of cDNA in the IgG condition). Red asterisks show samples excluded from further analysis because of a significantly lower number of unique counts and/or high ratio of unique reads over total reads compared to other samples.
c, Heatmap of correlation matrix for read coverage of binned genomics regions for each NPC, neuron, and non-crosslinked sample. The colored scale depicts the correlation values which are also indicated in each square. The y-axis on the left side shows the hierarchical clustering of the samples.
d, Metagene plot showing m6A residue distribution in NPCs. Embedded - consensus motif with the most significant p-value, as identified by HOMER motif discovery tools.
e, Venn diagram representing the overlap between m6A sites identified in neurons and a meta-analysis of a set of published RNA methylation datasets (see methods section).
f, Relative abundance levels of different proteins detected by MS in NPCs and neurons treated with siRNA CTRL. Expression was normalized based on the summed abundances of all proteins per sample, n=2 independent iPSC lines (biological replicates).
g, Boxplots of log2 FC (z-scored) from differentially expressed proteins in IMP1 siRNA (siIMP1) vs non-targeting control (siCTRL) neurons binned based on the number of IMP1- m6A sites on the corresponding RNA. Number of IMP1-m6a peaks per transcripts was obtained by overlapping the miCLIP and IMP1 iCLIP. For MS, n=3 independent iPSC lines for each condition (biological replicates); for iCLIP, n=3 technical replicates from 3 independent iPSC lines; miCLIP, n=2 technical replicates from 3 independent iPSC lines + 1 replicate from 1 independent iPSC line (biological replicates). The reported p-values were calculated using a two-sided Mann–Whitney U test comparing 1-2 peaks, 3-5 peaks or 6 and + peaks to 0 peaks. Data presented as boxplots where the center line is the median; limits are the interquartile range; and whiskers correspond to 1.5 times the interquartile range. Outliers are not displayed for clarity.

h, Table displaying 1) the proportion of targets or peaks bound by IMP1 via an m6A site, relative to the total number of targets or peaks bound by IMP1, and 2) the proportion of targets or peaks bound by IMP1 through an m6A site that are downregulated at the protein level following IMP1 knockdown, relative to all targets or peaks bound by IMP1 through m6A sites. Source data are provided as a Source Data file.
