# Supplementary material for: m6a methylation orchestrates IMP1 regulation of microtubules during human neuronal differentiation
Source: Nat Commun. 2024 Jun 6;15:4819. doi: 10.1038/s41467-024-49139-7 (PMC11156911; doi:10.1038/s41467-024-49139-7)
Supplement: Supplementary file 3 — Reporting Summary [file 41467_2024_49139_MOESM3_ESM.pdf]

nature portfolio

Corresponding author(s): Andres Ramos, Rickie Patani

Last updated by author(s): 25/04/24

Reporting Summary

Nature Portfolio wishes to improve the reproducibility of the work that we publish. This form provides structure for consistency and transparency in reporting. For further information on Nature Portfolio policies, see our Editorial Policies and the Editorial Policy Checklist.

Statistics

For all statistical analyses, confirm that the following items are present in the figure legend, table legend, main text, or Methods section.

n/a	Confirmed	
☐	☒	The exact sample size (*n*) for each experimental group/condition, given as a discrete number and unit of measurement
☐	☒	A statement on whether measurements were taken from distinct samples or whether the same sample was measured repeatedly
☐	☒	The statistical test(s) used AND whether they are one- or two-sided<br>*Only common tests should be described solely by name; describe more complex techniques in the Methods section.*
☐	☒	A description of all covariates tested
☐	☒	A description of any assumptions or corrections, such as tests of normality and adjustment for multiple comparisons
☐	☒	A full description of the statistical parameters including central tendency (e.g. means) or other basic estimates (e.g. regression coefficient) AND variation (e.g. standard deviation) or associated estimates of uncertainty (e.g. confidence intervals)
☐	☒	For null hypothesis testing, the test statistic (e.g. *F*, *t*, *r*) with confidence intervals, effect sizes, degrees of freedom and *P* value noted<br>*Give P values as exact values whenever suitable.*
☒	☐	For Bayesian analysis, information on the choice of priors and Markov chain Monte Carlo settings
☒	☐	For hierarchical and complex designs, identification of the appropriate level for tests and full reporting of outcomes
☐	☒	Estimates of effect sizes (e.g. Cohen's *d*, Pearson's *r*), indicating how they were calculated

Our web collection on statistics for biologists contains articles on many of the points above.

Software and code

Policy information about availability of computer code

Data collection	Immunofluorescence: Zeiss LSM 880 inverted confocal microscope, VT-iSIM<br>qPCR: QuantStudio 6 Flex Real-Time PCR System<br>Sequencing: Hiseq 4000 sequencer<br>RNA quality check: NanoDrop 2000, Agilent 2100 Bioanalyser<br>Signal scanning (WB and CLIP): LI-COR Odyssey CLx and Image Studio software v5.5.<br>Mass Spectrometry: Dionex UltiMate 3000 HPLC system, Dionex UltiMate 3000 UHPLC system coupled with the Orbitrap Lumos Mass Spectrometer<br>Luciferase assay: Ensight Plate Multimode Reader
Data analysis	Data organisation and processing: Microsoft Excel v16.6, GraphPad Prism v7 and 10, R v4.1.2<br>Image analysis and quantification: Fiji/ImageJ v2.3.0<br>NGS analysis: iCount v2.30.0, iMaps (Konig et al., 2010), Flexbar v2.5, pyCRAC v1.3.2, STAR aligner v2.6.1, Integrative Genome Viewer v2.8.0, Deseq2 v1.34.0, Bedtools v2.3.0.0, BEDOPS 2.4.39, DeepTool v2.0, FastQC v0.11.7, TrimGalore v0.6.5, samtools v1.9, clipplotr (https://github.com/ulelab/clipplotr), R v4.1.2, CTK package v1.1.4 (https://zhanglab.c2b2.columbia.edu/index.php/CTK_Documentation), Lift Genome Annotations (https://genome.ucsc.edu/cgi-bin/hgLiftOver)<br>GO analysis: PANTHER Classification System v16.0 (http://www.pantherdb.org), REVIGO v1.8.1 (Reduce and Visualize Gene Ontology) (Supek et al., 2011)<br>Protein-protein interaction: STRING v11.5 (Search Tool for the Retrieval of Interacting Genes/Proteins)<br>Motif Analysis: HOMER vhomer2 (Hypergeometric Optimization of Motif EnRichment)

Mass spectrometry: Proteome Discoverer v2.4
Metagene: MetaPlotR pipeline (https://github.com/olarerin/metaPlotR)

For manuscripts utilizing custom algorithms or software that are central to the research but not yet described in published literature, software must be made available to editors and reviewers. We strongly encourage code deposition in a community repository (e.g. GitHub). See the Nature Portfolio guidelines for submitting code & software for further information.

Data

Policy information about availability of data

All manuscripts must include a data availability statement. This statement should provide the following information, where applicable:

- Accession codes, unique identifiers, or web links for publicly available datasets
- A description of any restrictions on data availability
- For clinical datasets or third party data, please ensure that the statement adheres to our policy

The RNA-seq, iCLIP and miCLIP data generated for this paper have been deposited in NCBI Gene Expression Omnibus (GEO) under accession number GSE203571 [https://www.ncbi.nlm.nih.gov/geo/query/acc.cgi?acc=GSE203571]. RNA-seq data used to estimate the mRNA expression level in NPCs and neurons were obtained from GEO under accession number GSE98290 [https://www.ncbi.nlm.nih.gov/geo/query/acc.cgi?acc=GSE98290]. miCLIP and MeRIP-seq to generate nervous tissue m6A map (meta-analysis) were from GEO under accession number GSE106607 (https://www.ncbi.nlm.nih.gov/geo/query/acc.cgi?acc=GSE106607], GSE106423 [https://www.ncbi.nlm.nih.gov/geo/query/acc.cgi?acc=GSE106423], GSE71154 [https://www.ncbi.nlm.nih.gov/geo/query/acc.cgi?acc=GSE71154], GSE29714 [https://www.ncbi.nlm.nih.gov/geo/query/acc.cgi?acc=GSE29714], GSE37005 [https://www.ncbi.nlm.nih.gov/geo/query/acc.cgi?acc=GSE37005] and dataset from Yu et al. 2020 43. The mass spectrometry raw data have been deposited to PRIDE accession number PXD034341. Human reference genome hg38.p13 and v36 gtf annotation file from gencode were used. Crosslink values and transcript coordinates for metagene plots in neurons and NPCs have been deposited in Figshare [DOI: 10.6084/m9.figshare.25595715]. Source data are provided as a Source Data file. Uncropped gels are provided within the Source Data file. Data is presented as Source data or deposited in a public repository, whenever possible. Materials and any more specific information can be obtained directly from the authors, as standard.

Research involving human participants, their data, or biological material

Policy information about studies with human participants or human data. See also policy information about sex, gender (identity/presentation), and sexual orientation and race, ethnicity and racism.

Reporting on sex and gender	*Use the terms sex (biological attribute) and gender (shaped by social and cultural circumstances) carefully in order to avoid confusing both terms. Indicate if findings apply to only one sex or gender; describe whether sex and gender were considered in study design; whether sex and/or gender was determined based on self-reporting or assigned and methods used.* *Provide in the source data disaggregated sex and gender data, where this information has been collected, and if consent has been obtained for sharing of individual-level data; provide overall numbers in this Reporting Summary. Please state if this information has not been collected.* *Report sex- and gender-based analyses where performed, justify reasons for lack of sex- and gender-based analysis.*
Reporting on race, ethnicity, or other socially relevant groupings	*Please specify the socially constructed or socially relevant categorization variable(s) used in your manuscript and explain why they were used. Please note that such variables should not be used as proxies for other socially constructed/relevant variables (for example, race or ethnicity should not be used as a proxy for socioeconomic status).* *Provide clear definitions of the relevant terms used, how they were provided (by the participants/respondents, the researchers, or third parties), and the method(s) used to classify people into the different categories (e.g. self-report, census or administrative data, social media data, etc.)* *Please provide details about how you controlled for confounding variables in your analyses.*
Population characteristics	*Describe the covariate-relevant population characteristics of the human research participants (e.g. age, genotypic information, past and current diagnosis and treatment categories). If you filled out the behavioural & social sciences study design questions and have nothing to add here, write "See above."*
Recruitment	*Describe how participants were recruited. Outline any potential self-selection bias or other biases that may be present and how these are likely to impact results.*
Ethics oversight	*Identify the organization(s) that approved the study protocol.*

Note that full information on the approval of the study protocol must also be provided in the manuscript.

Field-specific reporting

Please select the one below that is the best fit for your research. If you are not sure, read the appropriate sections before making your selection.

☒ Life sciences ☐ Behavioural & social sciences ☐ Ecological, evolutionary & environmental sciences

For a reference copy of the document with all sections, see nature.com/documents/nr-reporting-summary-flat.pdf

Life sciences study design

All studies must disclose on these points even when the disclosure is negative.

Sample size	No statistical methods were used to predetermine sample sizes, which were based on published studies with similar approaches in the field – see Dawicki-McKenna et al, 2023, Schöndorf et al, 2014, Xu et al, 2024, Leoni et al 2023, Kodavati et al, 2024, Taylor et al, 2022, Altman et al 2021, all from Nature communications. Biological replicates used in various experiments in this study are as follows: RNAseq and MS experiments were performed on 3 biological replicates for neurons, and 2 for MS and 3 biological replicates for RNAseq for NPCs. iCLIP experiment was performed on 3 technical replicates from 3 biological replicates, miCLIP experiment was performed on 2 technical replicates from 4 biological replicates. Luciferase assay was performed on 8 replicates (4 independent experiments). For WB and IF experiments were performed on at least 2 biological replicates.
Data exclusions	Samples from iCLIP and miCLIP experiments with low quality as defined by a significantly lower number of unique reads compared to other samples were excluded. Data points (2 out of 8 for condition DCX Vector+IMP1 over expression+ siRNA CTRL and 1 out of 8 replicates for condition DCX Vector+IMP1 over expression + siRNA METTL3) were excluded because of pipetting errors in the luciferase assay.
Replication	All experiments were at least performed once in 2 independent iPSC lines per group (biological replicates). Number of biological and technical replicates are all detailed in the figure legends in this manuscript. All attempts to reproduce results were successful.
Randomization	No randomization was required since all experiments were performed on cellular populations. All experiments presented in this study involved unbiased analyses.
Blinding	Binding was not required because the results presented involved experimental procedures which could not be affected by the knowledge of sample identity. All analyses presented in this study used objective quantitative methods using experimental instrumentation or automatized procedure (including ICC quantifications)

Behavioural & social sciences study design

All studies must disclose on these points even when the disclosure is negative.

Study description	*Briefly describe the study type including whether data are quantitative, qualitative, or mixed-methods (e.g. qualitative cross-sectional, quantitative experimental, mixed-methods case study).*
Research sample	*State the research sample (e.g. Harvard university undergraduates, villagers in rural India) and provide relevant demographic information (e.g. age, sex) and indicate whether the sample is representative. Provide a rationale for the study sample chosen. For studies involving existing datasets, please describe the dataset and source.*
Sampling strategy	*Describe the sampling procedure (e.g. random, snowball, stratified, convenience). Describe the statistical methods that were used to predetermine sample size OR if no sample-size calculation was performed, describe how sample sizes were chosen and provide a rationale for why these sample sizes are sufficient. For qualitative data, please indicate whether data saturation was considered, and what criteria were used to decide that no further sampling was needed.*
Data collection	*Provide details about the data collection procedure, including the instruments or devices used to record the data (e.g. pen and paper, computer, eye tracker, video or audio equipment) whether anyone was present besides the participant(s) and the researcher, and whether the researcher was blind to experimental condition and/or the study hypothesis during data collection.*
Timing	*Indicate the start and stop dates of data collection. If there is a gap between collection periods, state the dates for each sample cohort.*
Data exclusions	*If no data were excluded from the analyses, state so OR if data were excluded, provide the exact number of exclusions and the rationale behind them, indicating whether exclusion criteria were pre-established.*
Non-participation	*State how many participants dropped out/declined participation and the reason(s) given OR provide response rate OR state that no participants dropped out/declined participation.*
Randomization	*If participants were not allocated into experimental groups, state so OR describe how participants were allocated to groups, and if allocation was not random, describe how covariates were controlled.*

Ecological, evolutionary & environmental sciences study design

All studies must disclose on these points even when the disclosure is negative.

Study description	*Briefly describe the study. For quantitative data include treatment factors and interactions, design structure (e.g. factorial, nested, hierarchical), nature and number of experimental units and replicates.*
Research sample	*Describe the research sample (e.g. a group of tagged Passer domesticus, all Stenocereus thurberi within Organ Pipe Cactus National Monument), and provide a rationale for the sample choice. When relevant, describe the organism taxa, source, sex, age range and any manipulations. State what population the sample is meant to represent when applicable. For studies involving existing datasets,*

describe the data and its source.

Sampling strategy	Note the sampling procedure. Describe the statistical methods that were used to predetermine sample size OR if no sample-size calculation was performed, describe how sample sizes were chosen and provide a rationale for why these sample sizes are sufficient.
Data collection	Describe the data collection procedure, including who recorded the data and how.
Timing and spatial scale	Indicate the start and stop dates of data collection, noting the frequency and periodicity of sampling and providing a rationale for these choices. If there is a gap between collection periods, state the dates for each sample cohort. Specify the spatial scale from which the data are taken
Data exclusions	If no data were excluded from the analyses, state so OR if data were excluded, describe the exclusions and the rationale behind them, indicating whether exclusion criteria were pre-established.
Reproducibility	Describe the measures taken to verify the reproducibility of experimental findings. For each experiment, note whether any attempts to repeat the experiment failed OR state that all attempts to repeat the experiment were successful.
Randomization	Describe how samples/organisms/participants were allocated into groups. If allocation was not random, describe how covariates were controlled. If this is not relevant to your study, explain why.
Blinding	Describe the extent of blinding used during data acquisition and analysis. If blinding was not possible, describe why OR explain why blinding was not relevant to your study.

Did the study involve field work? ☐ Yes ☐ No

Field work, collection and transport

Field conditions	Describe the study conditions for field work, providing relevant parameters (e.g. temperature, rainfall).
Location	State the location of the sampling or experiment, providing relevant parameters (e.g. latitude and longitude, elevation, water depth).
Access & import/export	Describe the efforts you have made to access habitats and to collect and import/export your samples in a responsible manner and in compliance with local, national and international laws, noting any permits that were obtained (give the name of the issuing authority, the date of issue, and any identifying information).
Disturbance	Describe any disturbance caused by the study and how it was minimized.

Reporting for specific materials, systems and methods

We require information from authors about some types of materials, experimental systems and methods used in many studies. Here, indicate whether each material, system or method listed is relevant to your study. If you are not sure if a list item applies to your research, read the appropriate section before selecting a response.

Materials & experimental systems

n/a	Involved in the study	
☐	☒	Antibodies
☐	☒	Eukaryotic cell lines
☒	☐	Palaeontology and archaeology
☒	☐	Animals and other organisms
☒	☐	Clinical data
☒	☐	Dual use research of concern
☒	☐	Plants

Methods

n/a	Involved in the study	
☒	☐	ChIP-seq
☒	☐	Flow cytometry
☒	☐	MRI-based neuroimaging

Antibodies

Antibodies used

rabbit anti-IMP1 (MBL, RN007P), polyclonal, dilution 1:1000 (WB), dilution 1:100 (IF), 5ug for iCLIP
rabbit anti-IgG (Proteintech, 30000-0-AP), polyclonal, 5ug for miCLIP and iCLIP
rabbit anti-GAPDH (Cell signalling 14C10), monoclonal, dilution 1:1000 (WB),
mouse anti-Actb (Sigma, A2228), monoclonal, dilution 1:1000 (WB),
rabbit anti-H3 (Abcam, ab201456), monoclonal, dilution 1:2000 (WB),
chicken anti-Homer-1 (Synaptic System, 160006), polyclonal, dilution 1:100 (IF)
mouse anti-Synaptotagmin1 (Synaptic System, 105011C3), monoclonal, dilution 1:100 (IF)
mouse anti-SMI-35 (BioLegend, 835603), monoclonal, dilution 1:100 (IF)
rabbit anti-MAP1B (Proteintech, 21633-1-AP), polyclonal, dilution 1:100 (IF)

mouse anti-MAP2 (Abcam, ab11267), monoclonal, 1:1000 (WB),
chicken anti-MAP2 (Abcam, ab5392), polyclonal, dilution 1:100 (IF)
chicken anti-beta III tubulin (Abcam, ab41489), polyclonal, dilution 1:100 (IF)
mouse anti-beta IV tubulin (Abcam, ab11315), monoclonal, dilution 1:100 (IF)
rabbit anti-DCX (Proteintech, 13925-1-AP), polyclonal, dilution 1:1000 (WB), dilution 1:100 (IF)
mouse anti-MAPT (Abcam, ab80579), monoclonal, dilution 1:100 (IF)
rabbit anti-CRIPT (Proteintech,11211-1-AP), polyclonal, dilution 1:100 (IF)
mouse anti-m6A (Abcam, ab151230), polyclonal, 5ug for miCLIP
rabbit anti-METTL3 (Proteintech, 15073-1-AP), polyclonal, 1:1000 (WB)
IRDye-800CW (Licor), dilution 1:15000
IRDye-680CW (Licor), dilution 1:15000
Donkey anti-rabbit IgG 568 (Thermo Fischer Scientific), dilution 1:1000 (IF)
Donkey anti-rabbit IgG 647 (Thermo Fischer Scientific), dilution 1:1000 (IF)
Donkey anti-mouse IgG 488 (Thermo Fischer Scientific), dilution 1:1000 (IF)
Goat anti-Chicken IgG 568 (Thermo Fischer Scientific), dilution 1:1000 (IF)
Goat anti-Chicken IgG 488 (Thermo Fischer Scientific), dilution 1:1000 (IF)
Goat anti-Chicken IgG 647 (Thermo Fischer Scientific), dilution 1:1000 (IF)

Validation

Antibodies in this study are commercially available and have been validated by KD/KO and/or by the manufacturer and have been used in previous studies. Details on each antibody can be found on corresponding website:
rabbit anti-IMP1 (MBL, RN007P), using shRNA KD (WB and IF), https://www.mblintl.com/products/rn007p/
rabbit anti-IgG (Proteintech, 30000-0-AP), cited in 64 publications on citeAb, https://www.ptglab.com/products/IgG-control-Antibody-30000-0-AP.html
rabbit anti-GAPDH (Cell signalling 14C10), cited in 7,661 publications on citeAb, https://www.cellsignal.com/products/primary-antibodies/gapdh-14c10-rabbit-mab/2118?gclid=CjwKCAjwwr6wBhBcEiwAfMEQs17tbhFA0sQ0zCej7IbfEGYEu-RVzjOQhu5x9tCBZwitxdHmxZ1pBBoCHPAQAvD_BwE&gclsrc=aw.ds
mouse anti-Actb (Sigma, A2228), cited in 2534 publications on citeAb, https://www.sigmaaldrich.com/GB/en/search/a2228?focus=products&page=1&perpage=30&sort=relevance&term=a2228&type=product
rabbit anti-H3 (Abcam, ab201456), cited in 21 publications on citeAb, https://www.abcam.com/products/primary-antibodies/histone-h3-antibody-epr17785-ab201456.html
chicken anti-Homer-1 (Synaptic System, 160006), cited in 116 publications on citeAb, https://sysy.com/product/160006
mouse anti-Synaptotagmin1 (Synaptic System, 105011), cited in 96 publications on citeAb, https://sysy.com/product/105011, KO validated,
mouse anti-SMI-35 (Biolegend, 835603), https://www.biolegend.com/de-at/products/purified-anti-neurofilament-h-m-nf-h-nf-m-hypophosphorylated-antibody-12722
rabbit anti-MAP1B (Proteintech, 21633-1-AP), cited in 118 publications on citeAb, manufacturer KD/KO validation, https://www.ptglab.com/products/MAP1B-Antibody-21633-1-AP.htm
mouse anti-MAP2 (Abcam, ab11267), cited in 151 publications on citeAb, https://www.abcam.com/products/primary-antibodies/map2-antibody-hm-2-ab11267.html
chicken anti-MAP2 (Abcam, ab5392), cited in 643 publications on citeAb, https://www.abcam.com/products/primary-antibodies/map2-antibody-ab5392.html
chicken anti-beta III tubulin (Abcam, ab41489), cited in 55 publications on citeAb, https://www.abcam.com/products/primaryantibodies/beta-iii-tubulin-antibody-ab41489.html
mouse anti-beta IV tubulin (Abcam, ab11315), cited in 39 publications on citeAb, https://www.abcam.com/products/primaryantibodies/beta-iv-tubulin-antibody-ons1a6-ab11315.html
rabbit anti-DCX (Proteintech, 13925-1-AP), cited in 9 publications on citeAb, https://www.ptglab.com/products/DCX-Antibody-13925-1-AP.htm
mouse anti-MAPT (Abcam, ab80579), cited in 119 publications on citeAb, https://www.abcam.com/products/primary-antibodies/tauantibody-tau-5-bsa-and-azide-free-ab80579.html
rabbit anti-CRIPT (Proteintech,11211-1-AP), manufacturer KD/KO validation, https://www.ptglab.com/products/CRIPT-Antibody-11211-1-AP.htm
mouse anti-m6A (Abcam, ab151230), cited in 94 publications on citeAb, https://www.abcam.com/products/primary-antibodies/n6-methyladenosine-m6a-antibody-ab151230.html
rabbit anti-METTL3 (Proteintech, 15073-1-AP) cited in 188 publications on citeAb, manufacturer KD/KO validation, https://www.ptglab.com/products/METTL3-Antibody-15073-1-AP.htm

Eukaryotic cell lines

Policy information about cell lines and Sex and Gender in Research

Cell line source(s)

- hiPSC cells were obtained from healthy controls subjects. Experimental protocols were carried out according to approved regulations and guidelines by UCLH's National Hospital for Neurology and Neurosurgery and UCL's Institute of Neurology joint research ethics committee (09/0272). Neuronal progenitors and neurons were derived from hiPSC using differentiation protocol (Hall et all). Clone1 and clone 4 were made in house (Hall et al). Clone 2, 3, 5 and 6 are commercially available – clone 2, Coriell, identifier ND41866*C; clone 3, Thermo Fisher, identifier A18945; clone 5, Cedars-Sinai, identifier CS0002iCTR-nxx; and clone 6, NIH CRM, identifier CRMi003-A. Four male (C1, C2, C5, C6) and two females clones (C3, C4) were used in this study. It is important to note, that comparisons in this study were performed on groups containing matched isogenic cell lines (NPCs vs neurons) and/or matched cells upon treatment (siRNA).
- HeLa cells were obtained from Cell Services at the Francis Crick Institute . Cells were authenticated using STR PCR profiling

Authentication

Authentication was done by STR PCR genotyping and karyotyping

Mycoplasma contamination

Cells were routinellsly tested for mycosplasma and no contamination was detected at any point in this study

Commonly misidentified lines (See ICLAC register)	No commonly misidentified cell lines were used in this study

Palaeontology and Archaeology

Specimen provenance	*Provide provenance information for specimens and describe permits that were obtained for the work (including the name of the issuing authority, the date of issue, and any identifying information). Permits should encompass collection and, where applicable, export.*
Specimen deposition	*Indicate where the specimens have been deposited to permit free access by other researchers.*
Dating methods	*If new dates are provided, describe how they were obtained (e.g. collection, storage, sample pretreatment and measurement), where they were obtained (i.e. lab name), the calibration program and the protocol for quality assurance OR state that no new dates are provided.*

☐ Tick this box to confirm that the raw and calibrated dates are available in the paper or in Supplementary Information.

Ethics oversight	*Identify the organization(s) that approved or provided guidance on the study protocol, OR state that no ethical approval or guidance was required and explain why not.*

Note that full information on the approval of the study protocol must also be provided in the manuscript.

Animals and other research organisms

Policy information about studies involving animals; ARRIVE guidelines recommended for reporting animal research, and Sex and Gender in Research

Laboratory animals	*For laboratory animals, report species, strain and age OR state that the study did not involve laboratory animals.*
Wild animals	*Provide details on animals observed in or captured in the field; report species and age where possible. Describe how animals were caught and transported and what happened to captive animals after the study (if killed, explain why and describe method; if released, say where and when) OR state that the study did not involve wild animals.*
Reporting on sex	*Indicate if findings apply to only one sex; describe whether sex was considered in study design, methods used for assigning sex. Provide data disaggregated for sex where this information has been collected in the source data as appropriate; provide overall numbers in this Reporting Summary. Please state if this information has not been collected. Report sex-based analyses where performed, justify reasons for lack of sex-based analysis.*
Field-collected samples	*For laboratory work with field-collected samples, describe all relevant parameters such as housing, maintenance, temperature, photoperiod and end-of-experiment protocol OR state that the study did not involve samples collected from the field.*
Ethics oversight	*Identify the organization(s) that approved or provided guidance on the study protocol, OR state that no ethical approval or guidance was required and explain why not.*

Note that full information on the approval of the study protocol must also be provided in the manuscript.

Clinical data

Policy information about clinical studies

All manuscripts should comply with the ICMJE guidelines for publication of clinical research and a completed CONSORT checklist must be included with all submissions.

Clinical trial registration	*Provide the trial registration number from ClinicalTrials.gov or an equivalent agency.*
Study protocol	*Note where the full trial protocol can be accessed OR if not available, explain why.*
Data collection	*Describe the settings and locales of data collection, noting the time periods of recruitment and data collection.*
Outcomes	*Describe how you pre-defined primary and secondary outcome measures and how you assessed these measures.*

Dual use research of concern

Policy information about dual use research of concern

Hazards

Could the accidental, deliberate or reckless misuse of agents or technologies generated in the work, or the application of information presented in the manuscript, pose a threat to:

No	Yes	
☐	☐	Public health
☐	☐	National security
☐	☐	Crops and/or livestock
☐	☐	Ecosystems
☐	☐	Any other significant area

Experiments of concern

Does the work involve any of these experiments of concern:

No	Yes	
☐	☐	Demonstrate how to render a vaccine ineffective
☐	☐	Confer resistance to therapeutically useful antibiotics or antiviral agents
☐	☐	Enhance the virulence of a pathogen or render a nonpathogen virulent
☐	☐	Increase transmissibility of a pathogen
☐	☐	Alter the host range of a pathogen
☐	☐	Enable evasion of diagnostic/detection modalities
☐	☐	Enable the weaponization of a biological agent or toxin
☐	☐	Any other potentially harmful combination of experiments and agents

Plants

Seed stocks	*Report on the source of all seed stocks or other plant material used. If applicable, state the seed stock centre and catalogue number. If plant specimens were collected from the field, describe the collection location, date and sampling procedures.*
Novel plant genotypes	*Describe the methods by which all novel plant genotypes were produced. This includes those generated by transgenic approaches, gene editing, chemical/radiation-based mutagenesis and hybridization. For transgenic lines, describe the transformation method, the number of independent lines analyzed and the generation upon which experiments were performed. For gene-edited lines, describe the editor used, the endogenous sequence targeted for editing, the targeting guide RNA sequence (if applicable) and how the editor was applied.*
Authentication	*Describe any authentication procedures for each seed stock used or novel genotype generated. Describe any experiments used to assess the effect of a mutation and, where applicable, how potential secondary effects (e.g. second site T-DNA insertions, mosiacism, off-target gene editing) were examined.*

ChIP-seq

Data deposition

☐ Confirm that both raw and final processed data have been deposited in a public database such as GEO.

☐ Confirm that you have deposited or provided access to graph files (e.g. BED files) for the called peaks.

Data access links<br>*May remain private before publication.*	*For "Initial submission" or "Revised version" documents, provide reviewer access links. For your "Final submission" document, provide a link to the deposited data.*
Files in database submission	*Provide a list of all files available in the database submission.*
Genome browser session<br>(e.g. UCSC)	*Provide a link to an anonymized genome browser session for "Initial submission" and "Revised version" documents only, to enable peer review. Write "no longer applicable" for "Final submission" documents.*

Methodology

Replicates	*Describe the experimental replicates, specifying number, type and replicate agreement.*
Sequencing depth	*Describe the sequencing depth for each experiment, providing the total number of reads, uniquely mapped reads, length of reads and whether they were paired- or single-end.*
Antibodies	*Describe the antibodies used for the ChIP-seq experiments; as applicable, provide supplier name, catalog number, clone name, and lot number.*
Peak calling parameters	*Specify the command line program and parameters used for read mapping and peak calling, including the ChIP, control and index files used.*

Data quality | *Describe the methods used to ensure data quality in full detail, including how many peaks are at FDR 5% and above 5-fold enrichment.*

Software | *Describe the software used to collect and analyze the ChIP-seq data. For custom code that has been deposited into a community repository, provide accession details.*

Flow Cytometry

Plots

Confirm that:

- [ ] The axis labels state the marker and fluorochrome used (e.g. CD4-FITC).
- [ ] The axis scales are clearly visible. Include numbers along axes only for bottom left plot of group (a 'group' is an analysis of identical markers).
- [ ] All plots are contour plots with outliers or pseudocolor plots.
- [ ] A numerical value for number of cells or percentage (with statistics) is provided.

Methodology

Sample preparation | *Describe the sample preparation, detailing the biological source of the cells and any tissue processing steps used.*

Instrument | *Identify the instrument used for data collection, specifying make and model number.*

Software | *Describe the software used to collect and analyze the flow cytometry data. For custom code that has been deposited into a community repository, provide accession details.*

Cell population abundance | *Describe the abundance of the relevant cell populations within post-sort fractions, providing details on the purity of the samples and how it was determined.*

Gating strategy | *Describe the gating strategy used for all relevant experiments, specifying the preliminary FSC/SSC gates of the starting cell population, indicating where boundaries between "positive" and "negative" staining cell populations are defined.*

- [ ] Tick this box to confirm that a figure exemplifying the gating strategy is provided in the Supplementary Information.

Magnetic resonance imaging

Experimental design

Design type | *Indicate task or resting state; event-related or block design.*

Design specifications | *Specify the number of blocks, trials or experimental units per session and/or subject, and specify the length of each trial or block (if trials are blocked) and interval between trials.*

Behavioral performance measures | *State number and/or type of variables recorded (e.g. correct button press, response time) and what statistics were used to establish that the subjects were performing the task as expected (e.g. mean, range, and/or standard deviation across subjects).*

Acquisition

Imaging type(s) | *Specify: functional, structural, diffusion, perfusion.*

Field strength | *Specify in Tesla*

Sequence & imaging parameters | *Specify the pulse sequence type (gradient echo, spin echo, etc.), imaging type (EPI, spiral, etc.), field of view, matrix size, slice thickness, orientation and TE/TR/flip angle.*

Area of acquisition | *State whether a whole brain scan was used OR define the area of acquisition, describing how the region was determined.*

Diffusion MRI ☐ Used ☐ Not used

Preprocessing

Preprocessing software | *Provide detail on software version and revision number and on specific parameters (model/functions, brain extraction, segmentation, smoothing kernel size, etc.).*

Normalization | *If data were normalized/standardized, describe the approach(es): specify linear or non-linear and define image types used for transformation OR indicate that data were not normalized and explain rationale for lack of normalization.*

Normalization template | *Describe the template used for normalization/transformation, specifying subject space or group standardized space (e.g. original Talairach, MNI305, ICBM152) OR indicate that the data were not normalized.*

Noise and artifact removal | *Describe your procedure(s) for artifact and structured noise removal, specifying motion parameters, tissue signals and physiological signals (heart rate, respiration).*

Volume censoring | *Define your software and/or method and criteria for volume censoring, and state the extent of such censoring.*

Statistical modeling & inference

Model type and settings | *Specify type (mass univariate, multivariate, RSA, predictive, etc.) and describe essential details of the model at the first and second levels (e.g. fixed, random or mixed effects; drift or auto-correlation).*

Effect(s) tested | *Define precise effect in terms of the task or stimulus conditions instead of psychological concepts and indicate whether ANOVA or factorial designs were used.*

Specify type of analysis: ☐ Whole brain ☐ ROI-based ☐ Both

Statistic type for inference | *Specify voxel-wise or cluster-wise and report all relevant parameters for cluster-wise methods.*

(See Eklund et al. 2016)

Correction | *Describe the type of correction and how it is obtained for multiple comparisons (e.g. FWE, FDR, permutation or Monte Carlo).*

Models & analysis

n/a	Involved in the study
☐	☐ Functional and/or effective connectivity
☐	☐ Graph analysis
☐	☐ Multivariate modeling or predictive analysis

Functional and/or effective connectivity | *Report the measures of dependence used and the model details (e.g. Pearson correlation, partial correlation, mutual information).*

Graph analysis | *Report the dependent variable and connectivity measure, specifying weighted graph or binarized graph, subject- or group-level, and the global and/or node summaries used (e.g. clustering coefficient, efficiency, etc.).*

Multivariate modeling and predictive analysis | *Specify independent variables, features extraction and dimension reduction, model, training and evaluation metrics.*
